# Severe dystonia, cerebellar atrophy, and cardiomyopathy likely caused by a missense mutation in *TOR1AIP1*

**DOI:** 10.1186/s13023-014-0174-9

**Published:** 2014-11-26

**Authors:** Imen Dorboz, Marie Coutelier, Anne T Bertrand, Jean-Hubert Caberg, Monique Elmaleh-Bergès, Jeanne Lainé, Giovanni Stevanin, Gisèle Bonne, Odile Boespflug-Tanguy, Laurent Servais

**Affiliations:** Inserm U1141, Université Paris Diderot-Sorbonne Paris Cité, DHU PROTECT, Paris, F-75019 France; Inserm, U1127, Paris, F-75013 France; CNRS, UMR 7225, Paris, 75013 France; Université Pierre et Marie Curie - Paris 6, UMR_S 1127, Institut du Cerveau et de la Moelle épinière, CHU Pitié-Salpêtrière, 75013 Paris, France; Laboratoire de Neurogénétique, Ecole Pratique des Hautes Etudes, Institut du Cerveau et de la Moelle épinière, CHU Pitié-Salpêtrière, 75013 Paris, France; Laboratoire de Génétique Humaine, Institut de Duve, UCL, 1200 Bruxelles, Belgium; Inserm, U974, Paris, F-75013 France; Université Pierre et Marie Curie - Paris 6, UM 76; CNRS, UMR 7215; Institut de Myologie, Paris, F-75013 France; Service de génétique, CHU du Sart Tilman, Liège, Belgium; Service d’Imagerie Pédiatrique, Hôpital Robert Debré, 75019 Paris, France; Département de Physiologie, Université Pierre et Marie Curie - Paris 6, Site Pitié-Salpêtrière, Paris, F-75013 France; AP-HP, Groupe Hospitalier Pitié-Salpêtrière, U.F. Cardiogénétique et Myogénétique, Service de Biochimie Métabolique, Paris, F-75013 France; Service de neurologie pédiatrique et des maladies métaboliques, Hôpital Robert Debré, Assistance Publique des Hôpitaux de Paris, 75019 Paris, France; Centre de Référence des Maladies Neuromusculaires, Hôpital de La Citadelle, 4000 Liège, Belgium; Institut de Myologie, Bâtiment Babinski, Hôpital de La Pitié Salpêtrière, 48/83 boulevard de l’Hôpital, 75013 Paris, France

**Keywords:** Dystonia, Cerebellar atrophy, Torsin, Nuclear membrane

## Abstract

**Background:**

Dystonia, cerebellar atrophy, and cardiomyopathy constitute a rare association.

**Methods:**

We used homozygosity mapping and whole exome sequencing to determine the mutation, western blot and immunolabelling on cultured fibroblasts to demonstrate the lower expression and the mislocalization of the protein.

**Results:**

We report on a boy born from consanguineous healthy parents, who presented at three years of age with rapidly progressing dystonia, progressive cerebellar atrophy, and dilated cardiomyopathy. We identified regions of homozygosity and performed whole exome sequencing that revealed a homozygous missense mutation in *TOR1AIP1*. The mutation, absent in controls, results in a change of a highly conserved glutamic acid to alanine. *TOR1AIP1* encodes lamina-associated polypeptide 1 (LAP1), a transmembrane protein ubiquitously expressed in the inner nuclear membrane. LAP1 interacts with torsinA, the protein mutated in DYT1-dystonia. *In vitro* studies in fibroblasts of the patient revealed reduced expression of LAP1 and its mislocalization and aggregation in the endoplasmic reticulum as underlying pathogenic mechanisms.

**Conclusions and relevance:**

The pathogenic role of *TOR1AIP1* mutation is supported by a) the involvement of a highly conserved amino acid, b) the absence of the mutation in controls, c) the functional interaction of LAP1 with torsinA, and d) mislocalization of LAP1 in patient cells. Of note, cardiomyopathy has been reported in LAP1-null mice and in patients with the *TOR1AIP1* nonsense mutation. Other cases will help delineate the clinical spectrum of LAP1-related mutations.

**Electronic supplementary material:**

The online version of this article (doi:10.1186/s13023-014-0174-9) contains supplementary material, which is available to authorized users.

## Background

Dystonia, cerebellar atrophy and cardiomyopathy constitute a very rare association, observed in rare mitochondrial disease and organic acidemia. We report on a boy with early onset dystonia, for whom work-up performed in several institutions failed to reach a diagnosis.

## Methods

We performed homozygosity mapping in the patient by using the Human Omni2.5 array (Illumina), and whole-exome sequencing (IntegraGen), using the SureSelect V4 capture kit (Agilent) and the HighSeq2000 sequencer (Illumina) [1].

For cell cultures work up, skin fibroblasts were maintained in DMEM (Life Technologies) with 10% FBS and penicillin/streptomycin. Cells were either plated on glass coverslips for immunofluorescence or pelleted for protein analysis. Cells were imaged using an Olympus FV-1000 confocal microscope, and electron microscopy was performed as described [2,3].

For Protein extraction and immunoblotting, Cell pellets were lysed in Protein Lysis Buffer. Proteins (20 μg) were separated by SDS-PAGE and transferred to nitrocellulose. Membranes were blocked with blocking buffer and incubated with primary or secondary antibodies diluted in the same buffer [3] (secondary Alexa-conjugates antibodies: Life Technologies; HRP-conjugated antibodies: Jackson ImmunoResearch).

### Ethic statement

Written informed consent was obtained from the patient’s legal guardian(s) for gene analysis and publication of this case report and any accompanying images. A copy of the written consent is available for review by the Editor-in-Chief of this journal.

## Result and discussion

**Figure 1:**
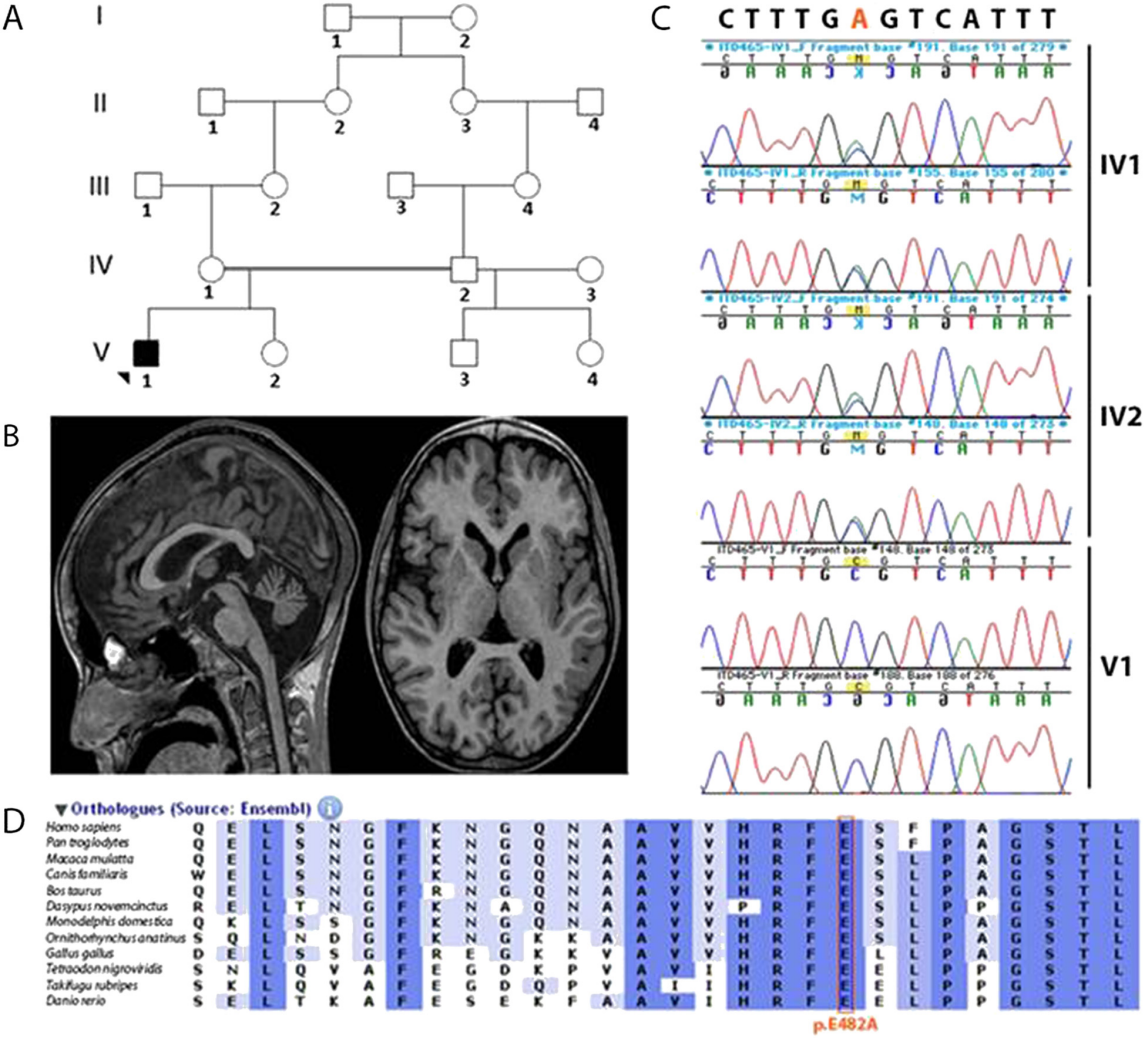
**Familial pedigree and brain MRI of the patient. A**. Familial pedigree. **B**. Sagittal 3DT1 gradient-echo image shows enlargement of the vermian fissures, which demonstrated cerebellar atrophy (mainly anterior). Reformatted axial 3DT1 gradient-echo image shows small lentiform nuclei compared to caudate nuclei volume. Slight enlargement of the lateral ventricles was also present. **C**. Sanger sequencing of *TOR1AIP1* shows a homozygous A to C variant at position 179,887,067 on chromosome 1 in the patient (V1). Both parents are heterozygous carriers (IV 1 and IV2). **D**. The mutated glutamic acid (surrounded by blue lines) is conserved across a broad range of species.

The boy was born after an uneventful pregnancy to healthy Moroccan parents who are second-degree related (Figure 1A). Familial history was irrelevant. Motor milestones were normal until the age of three, when the boy began to fall frequently and to develop progressive dystonia that first affected lower limbs and spread rapidly to upper limbs, trunk, and face. He lost ambulation at the age of 5. Dystonia was permanent, exacerbated by illness or stress, and by the age of 10 was completely generalized and extremely painful. The child spent most of the day and night crying and was unable to maintain a sufficient level of food and liquid intake (Additional file 1: Video S1). Severe contractures of the Achilles tendons and permanent feet deformations were noticed. Cerebellar maneuvers on upper limbs were normal. There was no ophthalmoplegia, ptosis, or pyramidal signs. Intelligence appeared normal. Medications, including paracetamol, opioids, benzodiazepines (diazepam, clonazepam, clobazam), L-Dopa, phenobarbitone, levetiracetam, piracetam, vigabatrin, propanolol, trihexyphenidyl hydrochloride, dantrolene, baclofen, carbamazepine, valproic acid, topiramate, chlorpromazine, amytriptiline, tetrabenazine, 1,3,5-benzenetriol, and magnesium sulfate, were tried unsuccessfully. The child’s pain was temporally relieved by intravenous chlorpromazine. Nabiximols led to a dramatic relief of pain and dystonia, and allowed the patient to re-acquire the ability to swallow, sit, and perform some voluntary movement (Additional file 2: Video S2). Attempts to wean the patient off medication led to rapid recurrence of pain. At the age of 14, a dilated cardiomyopathy was diagnosed, and ejection fraction rapidly decreased leading to death at the age of 17.

Prior to death, brain MRIs showed progressive global cerebellar atrophy (Figure 1B). Monovoxel MR spectroscopy of the left basal ganglia revealed a reduced NAA/Cr ratio indicative of neuronal loss without iron accumulation. Brain PET scans, electroencephalographic recordings, somatosensory evoked potentials, audition and fundus examination, electroneurography, liver and kidney echographies were unremarkable. Muscle biopsy, performed at the age of 6, revealed no abnormalities or biochemical deficits. Glucose, proteins, lactate, blood cell count, and neurotransmitters levels in the CSF were normal. Analyses for each of the following, performed at least once, were normal: blood cell count, ASAT, ALAT, CK, urea, creatinine, cholesterol, triglyceride, arterial lactate and pyruvate levels, ceruloplasmin, cupremia and cupruria, alpha fetoprotein, very long chain fatty acids and long chain fatty acids, biopterin, urine creatine and guanidinoacetate, amino acid (blood and urine) and organo acid (urine) chromatography, high-resolution caryotype, glucocerebrosidase, galactocerebrosidase, β-galactosidase, α-N-acetylgalacosaminidase, aryl sulfatase A, hexosaminidase A and B, α-glucosaminidase, β-glucuronidase, α-mannosidase, β-mannosidase, α-neuraminidase, acid sphingomyelinidase mucopolysaccharidoses and oligosaccharidoses, and sialotransferrin. No acanthocytosis was present on any of several blood smears.

No mutations were identified in *DYT1*, *PANK2*, *PLA2G6*, *SCA17*, or *APTX*, genes known to be involved in neurodegenerative diseases with cerebellar atrophy and/or dystonia. Given consanguinity in the family, we assumed a recessive mode of inheritance and predicted the causative variant would fall in a region of homozygosity.

Homozygosity mapping revealed six homozygous regions greater than 2 Mb. Whole exome sequencing produced 142 million reads, 98% of which could be aligned to the targeted sequence. Mean coverage of the targeted sequence was 77 fold. 56 687 SNVs and 7 265 indels were called. Only nine homozygous variants with frequency below 1% in internal (IntegraGen) and public databases (dbSNP 132; HapMap; 1,000 Genomes Project) were filtered out of these. Sequencing in the patient and in family members (primers available upon request) reduced the number of candidates to four genes, present at the homozygous state in the patient, and at the heterozygous state in the parents: *ALMS1*, *B3GALT1*, *ZNF804B*, and *TOR1AIP1*.

The A>C missense variant (c.1448A>C) at position 179,887,067 on chromosome 1q25.1-1q25.3 in *TOR1AIP1* (NM_015602), located in a 6.8-Mb homozygosity region, resulted in replacement of a highly conserved glutamic acid with alanine at amino acid 482 (GERP++ score 5.96; PhyloP score 2.285) (Figure 1C,D). Furthermore, pathogenicity predictions were deleterious in Align GVGD, Polyphen-2, SIFT, and MutationTaster analyses. On the contrary, the variants in *ALMS1* (NM_015120; c.2202T>A/p.S732R), *B3GALT1* (c.192A>T/p.E64D, NM_020981) and *ZNF804B* (c3118C>A/p.L1040I, NM_181646), were predicted to be benign by at least three of the above-mentioned programs. GERP++ and PhyloP scores were lower for the ZNF804B variant (GERP++ score 4.15, PhyloP score 1.467), and even negative for the *ALMS1* and *B3GALT1* variants. There was thus a strong bioinformatic convergence towards the pathogenic character of the *TOR1AIP1* variant only. In addition, the phenotype of this patient was divergent from that of Alström syndrome (OMIM #203800) patients who have mutations in *ALMS1. TOR1AIP1* encodes LAP1, a type II transmembrane protein. LAP1 interacts with torsinA (encoded by *TOR1A* gene), which is mutated in autosomal dominant dystonia (DYT1; OMIM #12810) [4]. The amino acid mutated in our patient is located in the luminal domain, which interacts with torsinA. This domain is common to the three isoforms and has significant homology with LULL1, another protein that interacts with torsinA. This variant was not observed in any of 100 ethnically matched controls and was absent from >6500 exomes at the Exome Variant Server.

**Figure 2:**
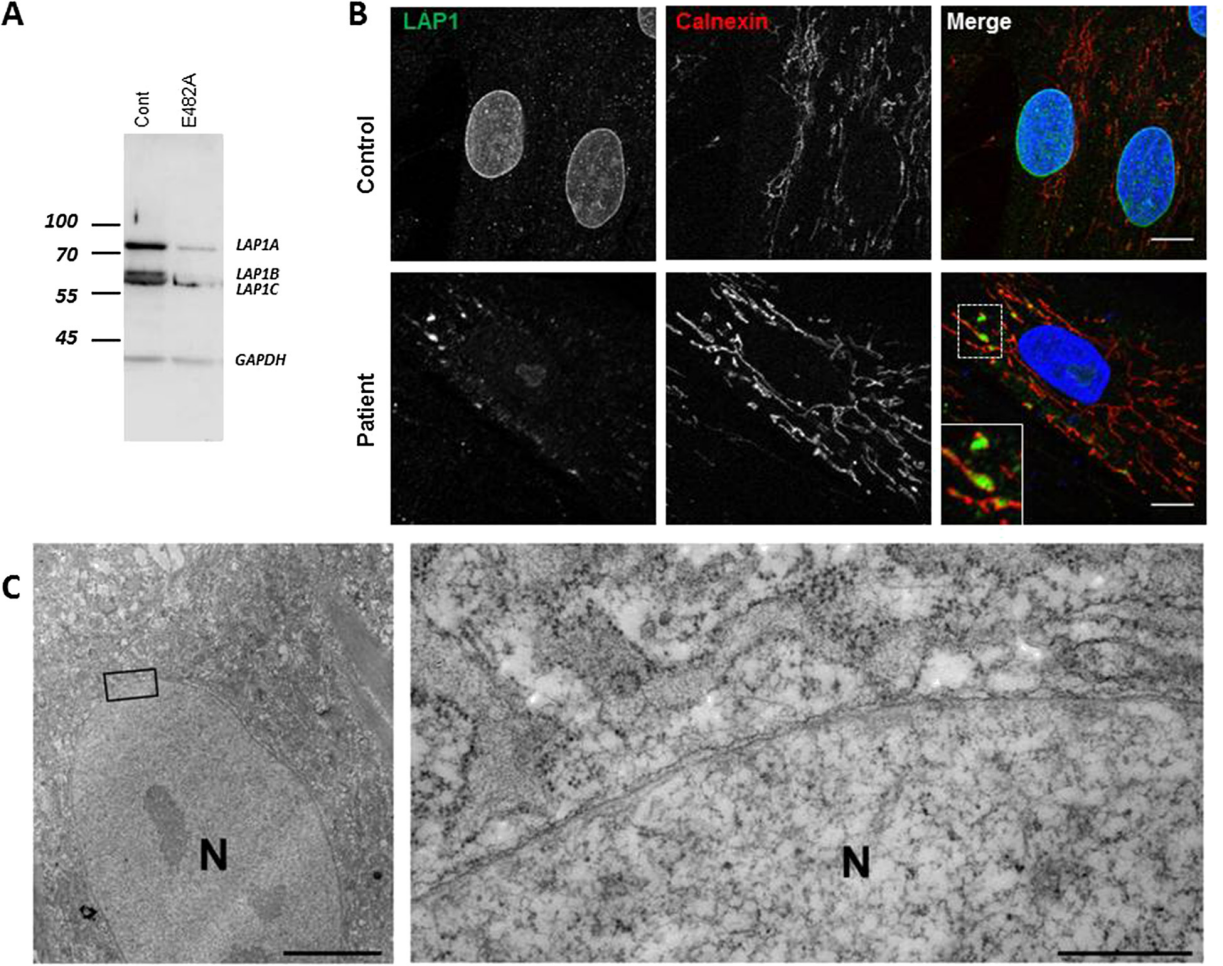
**Sub-expression and mislocalization of LAP1. A**. Three bands were observed using anti-LAP1 antibody in control fibroblasts. Fibroblasts from the patient had significantly less of the larger isoforms, whereas the expression of the shorter isoform was less affected (LAP1 antibodies: courtesy of Dr. W.T. Dauer, anti-GAPDH: Santa Cruz Biotechnology; Olympus FV-1000 confocal microscope). **B**. Fibroblasts from patient show a strong reduction or total absence of LAP1 at the nuclear envelope. A faint staining for LAP1 was observed at the ER (stained with calnexin antibodies, Santa Cruz Biotechnology; secondary Alexa-conjugates antibodies, Life Technologies; HRP-conjugated antibodies: Jackson ImmunoResearch), and strong staining was observed in some regions (see magnification in the inset). Scale bar: 10 μm. **C**. Electron micrograph of patient fibroblast did not reveal alterations at the nuclear envelope. Scale bar: 5 μm; inset: 500 nm. Imaging as described before [3].

To gain insight into the pathogenicity of the *TOR1AIP1* mutation, we evaluated primary skin fibroblasts from the patient. By western blot, a strong reduction in the expression of LAP1 isoforms was observed relative to control cells (Figure 2A). Immunolabeling revealed a significant reduction in LAP1 staining in the nuclear envelope of patient cells (Figure 2B). Although the endoplasmic reticulum was generally faintly stained, some areas showed accumulation of LAP1 (Figure 2B), indicating mislocalization of the mutated LAP1. No defects in A-type or B-type lamins, SUN1, SUN2, or nesprin-1 or 2 protein localization were observed (data not shown). Similarly, no blebs [4] in nuclear envelopes were observed by electron microscopy in patient cells (Figure 2C). TorsinA is not normally expressed in fibroblasts, so we were not able to determine if this protein was mislocalized.

We have searched *TOR1AIP1* mutation in 10 additional patients with dystonia during childhood: Five with primary dystonia associated with cerebellar atrophy and 5 with primary cerebellar ataxia with progressive cerebellar atrophy and severe dystonia. We did not find mutations, which seems to indicate that *TOR1AIP1* mutation is not a common cause of dystonia of unknown origin.

To our knowledge, cerebellar atrophy, dystonia, and dilated cardiomyopathy are very rarely associated, except in some mitochondrial disease or organic acidemia. Our patient did not suffer from organic acidemia and mitochondrial disorders were unlikely given normal blood and CSF lactate levels, NMR spectroscopy, and muscular biopsy. Cerebellar atrophy and dystonia are observed in neurodegenerative diseases, such as Wilson disease, acanthocytosis, gangliosidosis [5], and Niemann Pick type C [6] and in some cases of spinocerebellar ataxias, particularly SCA17 [7]. These were ruled out in the patient. *TUBB4A* mutations have been recently involved in patients with dystonia, cerebellar and basal ganglia atrophy and hypomyelinating leukodystrophies (HABC syndrome) [8]. In addition to the absence of white matter abnormalities in our case, exome analysis ruled out *TUBB4A* mutations. In the clinical work up, other causes of dystonia (such as *DYT1* and *PANK2* mutation) or cerebellar atrophy (such as *PLA2G6* mutation) were also ruled out. Much older patients who have presented with the association of a less severe dystonia and a cerebellar atrophy have been described [9]. Extreme painfull dystonia was one of the primary clinical features observed when we first evaluated the child at the age of 11. Absence of effect of any usual oral medications tried by us and others, and the dramatic improvement upon administration of nabiximols, a cannabinoid derived from cannabis plant [10], remains very enigmatic but as been reported in adults with central pain and paroxysmal dystonia [11].

LAP1, encoded by *TOR1AIP1*, induces the ATPase activity of torsinA [12], the protein mutated in DYT1, an autosomal dominant dystonia with purely neurological phenotype. LAP1 is the only protein with such function at the nuclear envelope described so far, and hence its mislocalization likely leads to a dramatic loss of function of torsinA and probably of other torsin family proteins [12]. Mice lacking LAP1 have abnormal nuclear membranes that form blebs visible by electron microscopy in all cell types [4]. In mice lacking torsinA, blebs are only present in neurons, probably because of the high level of torsinB in non-neuronal cells [13]. Such blebs were not observed in patient’s fibroblasts. The mutated LAP1 expressed by the patient may retain some functions required for the structure of the nuclear envelope. Our case appears much more severe than DYT1, not only because of the severity of dystonia itself, but because of the cardiac involvement, similar to the phenotype of *Tor1a*^∆*E*/∆*E*^ mice [4] and with disorders of nuclear membrane such as laminopathies [3]. Recently, cardiomyopathy has been demonstrated in a tissue-specific mutant lacking LAP1 [14].

One Turkish family with a homozygous nonsense mutation was recently reported [15]. Interestingly, affected patients also present with a cardiomyopathy, although less severe than that observed in our patient, and associated with a limb-girdle muscular dystrophy.

## Conclusions

The present observation is unique, and, therefore, it cannot be completely ruled out that the phenotype is not related to a mutation in a region not covered by exome sequencing. However, we believe that the association between the previously unreported phenotype of the patient and the mutation is very likely. First, clinical evaluations failed to identify another likely cause. Second, the mutation affects a highly conserved amino acid, is predicted to be pathogenic, is absent in 100 ethnically matched controls and in >6500 exomes, and affects a protein that interacts with a protein involved in dystonia. Third, the patient phenotype correlates with that of mice lacking LAP1 [14], and functional explorations of patient cells revealed reduced LAP1 expression and mislocalization. It is known that adequate localization of LAP1 is crucial for its function [12]. Finally, another family has been reported with nonsense mutation leading to cardiomyopathy [15]. For these different reasons, we believe that sequencing of *TOR1AIP1* should be performed in patients with similar peculiar phenotypic associations.

## Availability of supporting data

All supporting data are available within the limits of patient’s confidentiality.

## Additional files

Additional file 1: Video S1. Severe and painful dystonia before starting nabiximols. Additional file 2: Video S2. Video taken by the parents after three months of nabiximols. The father asks the child to move his leg.
